# Arsenic trioxide synergistically promotes the antileukaemic activity of venetoclax by downregulating Mcl-1 in acute myeloid leukaemia cells

**DOI:** 10.1186/s40164-021-00221-6

**Published:** 2021-04-15

**Authors:** Hyunsoo Cho, Ji Eun Jang, Ju-In Eom, Hoi-Kyung Jeung, Haerim Chung, Jin Seok Kim, June-Won Cheong, Yoo Hong Min

**Affiliations:** 1 Division of Hematology, Department of Internal Medicine, Severance Hospital, Yonsei University College of Medicine, 50-1 Yonsei-ro, Seodaemun-gu, Seoul, 03722 Republic of Korea; 2 Avison Biomedical Research Center, Yonsei University College of Medicine, Seoul, 03722 Republic of Korea

**Keywords:** Acute myeloid leukaemia, Venetoclax, Arsenic trioxide, Apoptosis

## Abstract

**Background:**

The evasion of apoptosis through dysregulated Bcl-2 family members is a hallmark of leukaemia stem cells (LSCs) in acute myeloid leukaemia (AML). Therefore, targeting Bcl-2 with venetoclax has been suggested as an attractive strategy for inducing apoptosis in AML LSCs. However, the selective inhibition of Bcl-2 in AML often leads to upregulation of Mcl-1, another dominant anti-apoptotic Bcl-2 family protein conferring venetoclax resistance.

**Methods:**

We assessed the combined effect of venetoclax and arsenic trioxide (ATO) on leukaemic cell viability, apoptosis, combination index, and cell cycle in the human LSC-like KG1 and KG1a cells. The synergistic effect of venetoclax and ATO on apoptosis was also examined in primary CD34^+^ and CD34^+^CD38^−^ LSCs from the bone marrow (BM) of AML patients, and compared with those from healthy donors**.**

**Results:**

Venetoclax efficiently impaired cell viability and dose-dependently promoted apoptosis when combined with ATO; their synergism was aptly represented by the combination index. The combination of venetoclax and ATO impaired cell cycle progression by restricting cells within the sub-G1 phase and facilitating caspase-dependent apoptotic cell death associated with the loss of mitochondrial membrane potential, while sparing healthy BM haematopoietic stem cells. Mechanistically, ATO mitigated venetoclax-induced upregulation of Mcl-1 by the inhibition of AKT and ERK, along with activation of GSK-3β. This led to the Mcl-1 destabilisation, triggering Noxa and Bim to facilitate apoptosis and the consequent activation of the apoptosis executioner protein Bak. Moreover, the combination promoted phosphorylation of ATM, Chk2, p38, and H2AX, indicating an active DNA damage response.

**Conclusions:**

Our findings demonstrate the synergistic, preferential antileukaemic effects of venetoclax and ATO on LSCs, providing a rationale for preclinical and clinical trials by combining these agents already being used in clinical practice to treat acute leukaemia.

**Supplementary Information:**

The online version contains supplementary material available at 10.1186/s40164-021-00221-6.

## Background

Acute myeloid leukaemia (AML) is a heterogeneous haematological malignancy involving haematopoietic stem cells (HSCs) and progenitor cells [1, 2]. Although a substantial proportion of AML patients achieve a complete remission with intensive cytotoxic chemotherapy, most patients succumb to disease relapse [1–5]. AML relapse is considered to originate from leukaemia stem cells (LSCs), which are metabolically quiescent, capable of self-renewal, and responsible for chemotherapy resistance [6, 7]. Therefore, more effective therapeutic strategies targeting LSCs are required to improve the cure rate in AML.

Bcl-2 protein is frequently overexpressed in AML LSCs [8]; this overexpression is associated with chemotherapy resistance, culminating in dismal clinical outcomes [9]. In this regard, development and clinical trials of Bcl-2 inhibitors have been conducted during recent years [10]. Venetoclax (ABT-199) is a BH3 mimetic, which selectively inhibits Bcl-2 and stabilises proapoptotic proteins [11]. It was shown that venetoclax can induce LSC cytotoxicity while largely sparing normal HSCs [12]. However, the biological and clinical actions of venetoclax employed as a single-agent have produced unsatisfactory results in AML [10]. Thus, the combination approach was developed, leading to approval for the use of venetoclax in combination with DNA methyltransferase inhibitors or low-dose cytarabine for elderly patients with newly diagnosed AML and those patients deemed unfit for conventional cytotoxic chemotherapy [13–17]. However, up to one-third of patients do not respond to these regimens [18, 19]. Besides, the majority of AML patients who achieve remission after receiving these combination therapies ultimately relapse, with a median duration of response of only about a year [18, 19]. Hence, there is an urgent requirement to elucidate the molecular mechanism underlying venetoclax resistance and develop novel combination strategies to target and overcome this resistance.

Mcl-1 is a critical anti-apoptotic protein that regulates cell survival in AML [20–22]. Mcl-1 upregulation coincides with episodes of relapse in chemotherapy-treated AML, emphasising the role of Mcl-1 in the development of drug resistance [23, 24]. Furthermore, emerging evidence suggests the upregulation of Mcl-1 protein as one of the mechanisms underlying acquired resistance to venetoclax treatment in AML [22–24]. Therefore, selective inhibitors of Mcl-1 are currently being investigated in preclinical and clinical studies on haematological malignancies, including AML [22]. However, the clinical development programme for a safe and selective Mcl-1 inhibitor has proven challenging so far. The large size, high lipophilicity, poor pharmacokinetic profile, limited cell membrane permeability, and shallow binding groove on Mcl-1 are the major hurdles impeding the development of Mcl-1 inhibitors [25]. Therefore, it is important to identify clinically available agents that interfere with Mcl-1 to augment the therapeutic efficacy of venetoclax in AML.

Arsenic trioxide (ATO) is a potent agent against acute promyelocytic leukaemia (APL) [26] and imparts a significant survival benefit in patients with relapsed APL [27]. Furthermore, recently acquired evidence favours the use of ATO with all-trans retinoic acid (ATRA) over anthracycline-based therapy as a first-line treatment option in newly diagnosed APL patients [28]. The toxicity profile of the ATO and ATRA combination appeared to be mild, with minimal myelosuppression and manageable adverse effects [28]. APL cell death by ATO is triggered by the generation of intracellular reactive oxygen species and induction of DNA damage response [29]. Indeed, ATO activates the intrinsic apoptotic pathway by lowering the mitochondrial membrane potential; additionally, ATO-induced apoptosis is associated with the downregulation of Bcl-2 and activation of proapoptotic executioner protein Bax [30]. Notably, ATO downregulates Mcl-1 expression in APL cells [31], although the underlying mechanism is not fully elucidated.

In the present study, we investigated whether the combination of venetoclax and ATO efficiently promotes apoptosis in AML LSC-like cells. Using LSC-like cell lines, as well as CD34^+^CD38^−^ primary AML cells and bone marrow (BM) cells from healthy donors, we demonstrated that the combination of venetoclax and ATO synergistically and selectively exhibits anti-AML activity in vitro, simultaneously sparing normal HSCs. We demonstrated that venetoclax-induced Mcl-1 upregulation is mitigated by ATO in AML LSC-like cells and revealed that downregulation of increased Mcl-1 levels is associated with the activation of GSK-3β. Our findings support a strategy for developing an effective and safe non-chemotherapeutic–based AML treatment regimen using a successful combination of venetoclax and ATO.

## Methods

### Cell lines and patient samples

This study was carried out in accordance with the Declaration of Helsinki and approved by the Institutional Review Board of Severance Hospital (Yonsei University College of Medicine, Seoul, Republic of Korea; 4-2010-0669). KG1 and KG1a human leukaemia cell lines were obtained from the American Type Culture Collection (Manassas, VA, USA). KG1 cells were cultured in Roswell Park Memorial Institute-1640 medium (Gibco, Thermo Fisher Scientific, Waltham, MA, USA), and KG1a cells were cultured in Iscove’s modified Dulbecco’s medium (Gibco). All media were supplemented with 10% foetal bovine serum, 100 U/mL penicillin, and 100 μg/mL streptomycin (Gibco) at 37 °C in a humidified environment under 5% CO_2_. Primary samples were obtained from the BM aspirates of AML patients at diagnosis (*n* = 4) and healthy donors who donated their BM aspirates for allogeneic haematopoietic stem cell transplantation (HSCT) (*n* = 4). To minimise the confounding effects specific to cytogenetic and/or molecular abnormalities, we utilised primary AML blasts only from cytogenetically normal AML patients without recurrent mutations. The clinical characteristics of AML patients at diagnosis or at relapse are summarised in Additional file 1: Table S1, S2. BM mononuclear cells (BMMCs) were isolated by Ficoll–Hypaque (GE Healthcare, Chicago, IL, USA) density gradient centrifugation. The patient cohort was registered at ClinicalTrials.gov (NCT02344966), and all patients and healthy donors provided written informed consent.

### Cell culture and treatment

A stock solution of venetoclax (Selleckchem, Houston, TX, USA) was prepared in dimethyl sulfoxide (DMSO), and serial dilutions were prepared in culture medium prior to each experiment. The final DMSO concentration was less than 0.2% (v/v) in all experiments. ATO (As_2_O_3_, Sigma-Aldrich, St. Louis, MO, USA) was dissolved in 1.65 M NaOH at 5 × 10^−2^ M to form a stock solution. The maximum concentration of NaOH in culture did not influence the growth of these cell lines. Logarithmically growing cells (1 × 10^5^ cells/mL) were exposed to different concentrations of venetoclax in the presence/absence of various concentrations of ATO. The pan-caspase inhibitor z-VAD-fmk (R&D Systems, Minneapolis, MN, USA) was added to the cells 2 h prior to venetoclax and ATO treatment.

### Reagents and antibodies

Rabbit polyclonal antibodies against caspase-9, caspase-3, poly(ADP-ribose) polymerase (PARP), phospho-p38, Mcl-1, phospho-Mcl-1 (T163), Bim, Bak, Bax, phospho-GSK-3β (S9), GSK-3β, phospho-AKT (S437), AKT, phospho-ERK (T202/Y204), phospho-ATR (S428), phospho-Chk1 (S317), phospho-Chk2 (T68), and p53 were purchased from Cell Signaling Technology (Danvers, MA, USA). The rabbit polyclonal antibody against Bcl-xL and mouse anti-Bcl-2 and anti-Noxa monoclonal antibodies were obtained from Santa Cruz Biotechnology (Dallas, TX, USA). The rabbit polyclonal antibody against phospho-Mcl-1 (S159) was obtained from BioVision (Milpitas, CA, USA). The rabbit polyclonal antibody against phospho-ATM (S1981) and mouse anti-phospho-H2AX (S139) monoclonal antibody were obtained from Abcam (Cambridge, UK). Horseradish peroxidase (HRP)-conjugated goat anti-rabbit IgG and HRP-conjugated goat anti-mouse IgG were from Cell Signaling Technology. The mouse anti-α–Tubulin monoclonal antibody was obtained from Merck Millipore (Burlington‚ MA, USA).

### Mcl-1 overexpression by transient transfection

The generation of Mcl-1 plasmid pcDNA3.1-Mcl-1 was described previously [32]. Briefly, a suspension of 2 × 10^6^ KG1a cells was transfected with 1 μg of pcDNA3.1 or pcDNA3.1-Mcl-1 using programme V-01 of the Amaxa Nucleofector device (Lonza Cologne GmbH, Cologne, Germany) according to the manufacturer’s instructions. Immediately after electroporation, the cells were resuspended in complete medium and incubated at 37 °C in a humidified atmosphere containing 5% CO_2_ for 48 h.

### Assessment of cell viability

Cells were seeded in 96-well plates (1 × 10^5^ cells/mL) and incubated overnight before treatment. After 48 h of treatment with the indicated concentrations of venetoclax in the presence/absence of ATO, 10 μL of the Cell Counting Kit-8 solution (Dojindo Molecular Technologies, Rockville, MD, USA) was added to each well. After incubation for 4 h, the absorbance at 450 nm was measured with a microplate reader (VersaMax, Molecular Devices, San Jose, CA, USA).

### Apoptosis assay

Apoptosis evaluation was performed by an Annexin V binding assay using LSR Fortessa flow cytometer (BD Biosciences, Franklin Lakes, NJ, USA). Cells were treated with various concentrations of venetoclax with or without ATO for 48 h and resuspended in Annexin V binding buffer. They were then incubated with Annexin V-FITC (BD Biosciences) and propidium iodide (PI) or 7-AAD (Beckman Coulter, Brea, CA, USA) for 15 min before flow cytometry analysis. To examine the apoptosis in the CD34^+^CD38^−^ cell fraction, cells were stained with anti-CD34-APC (BD Biosciences), anti-CD38-PE (BD Biosciences), and 7-AAD (Beckman Coulter) for 30 min. The labelled cells were subsequently resuspended in Annexin V binding buffer and incubated with Annexin V-FITC (BD Pharmingen) for 15 min before flow cytometry analysis. Data were analysed using the FACSuite software (BD Biosciences).

### Cell cycle analysis

Following each treatment cycle, cells were harvested, washed twice with PBS, and fixed in 70% ethanol at − 20 °C for 16 h. The fixed cells were washed twice with PBS and stained with PI for 15 min at 37 °C. Analysis of cells with sub-G1, G0/G1, S, and G2/M DNA content was performed using 10,000 cells on an LSR Fortessa flow cytometer (BD Biosciences). Data were analysed using the FACSuite software (BD Biosciences).

### Combination index

The combination effect was evaluated via quantitative analysis of dose–effect relationships based on the Chou-Talalay method as described previously [33]. A combination index (CI) value was calculated using the CalcuSyn software (Biosoft, San Francisco, CA, USA); CI < 1 was considered synergistic, CI = 1 was considered additive, and CI > 1 was considered antagonistic.

### Analysis of mitochondrial membrane potential

The mitochondrial membrane potential (MMP) was monitored using DiOC_6_, as described previously [34]. For each condition, 1 × 10^6^ cells were incubated with 1 mL of DePsipher solution (Trevigen, Gaithersburg, MD, USA), which uses a cationic dye (5,5′6,6′-tetrachloro-1,1′,3,3′tetraethylbenzimidazolylcarbocyanine iodide). After incubation for 20 min at 37 °C in a 5% CO_2_ incubator, cells were washed with 1 mL of pre-warmed 1X Reaction Buffer with Stabiliser Solution and subsequently analysed using an LSR Fortessa flow cytometer (488 nmargonlaser) and the FACSuite software (BD Biosciences).

### Western blot analysis

For protein extraction, cells were lysed with a radioimmunoprecipitation (RIPA) assay buffer (Thermo Fisher Scientific) supplemented with protease and phosphatase inhibitors (Roche, Basel, Switzerland). Cell lysates were centrifuged for 10 min at 4 °C and 13,000 rpm. Protein concentrations of the supernatants were quantitated using the detergent-insensitive Pierce BCA protein assay kit (Thermo Fisher Scientific). Lamni buffer was added to total protein lysates, and samples were denatured at 95 °C for 5 min. Aliquots of each protein lysate (10 μg) were subjected to SDS-PAGE. After electrophoresis, proteins were transferred to nitrocellulose membranes and blocked for 30 min with 5% bovine serum albumin in 0.1% Tween 20 in TBS (TBST). Primary antibodies were incubated overnight at 4 °C. After washing with TBST, membranes were incubated with the secondary peroxidase-coupled antibody for 1 h at room temperature and washed with TBST. The blots were visualised using enhanced chemiluminescence substrates (GE Healthcare). α-Tubulin was used as a loading control.

## Statistical analyses

All values are presented as mean ± standard deviation (s.d.). The statistical significance of differences between two groups was determined by the two-tailed unpaired *t*-test, one-way analysis of variance (ANOVA) followed by Tukey’s honest significant difference test with ranks for multiple-group comparison, or two-way ANOVA with Bonferroni post-hoc analysis. Statistical analyses were performed using GraphPad Prism 8 (GraphPad Software, San Diego, CA, USA). Statistical significance was considered at *P* < 0.05.

## Results

### The venetoclax and ATO combination synergistically promotes apoptosis in AML LSC-like cells

**Fig. 1:**
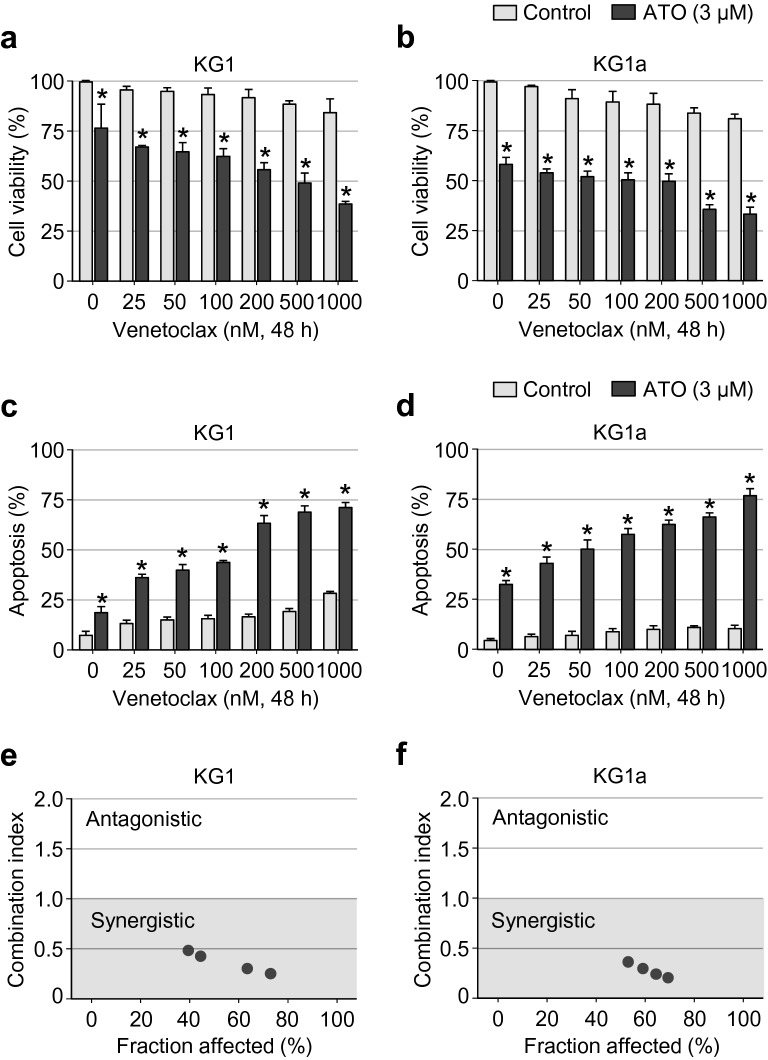
Venetoclax dose-dependently impairs cell viability and synergistically promotes apoptosis in KG1 and KG1a cells when in combination with ATO. **a**, **b**, Assessment of cell viability in KG1 (**a**) and KG1a (**b**) cells treated with the indicated concentration of venetoclax (0–1,000 nM) with or without ATO (3 µM) for 48 h. Cell viability was assessed by measuring the absorbance at 450 nm after incubating the treated cells for 4 h with Cell Counting Kit-8 solution (Dojindo). Values were obtained from three independent experiments, and horizontal bars indicate mean ± s.d. *P < 0.05 versus respective control by two-tailed Mann–Whitney *U* test. **c**, **d**, Summary data of the percentage of apoptotic fraction in KG1 (**c**) and KG1a (**d**) cells, as assessed by Annexin V and PI staining and flow cytometric analysis after treatment with the indicated concentration of venetoclax (0–1,000 nM) with or without ATO (3 µM) for 48 h. Values were obtained from three independent experiments, and horizontal bars indicate mean ± s.d. *P < 0.05 versus control treatment by two-tailed Mann–Whitney *U* test. **e**, **f**, Combination index of apoptotic cells after treatment with venetoclax and ATO in KG1 (**e**) and KG1a (**f**) cells. Values were obtained by median dose–effect analysis, and each dot indicates the value obtained from six independent experiments

To assess the combined antileukaemic effects of the venetoclax and ATO in AML LSC-like cells, we used the KG1 cell line, which is characterised by the high surface expression of CD34 but lacking CD38 (CD34^+^CD38^−^). The KG1a cell line, with similar phenotypic characteristics, was also included in the present study. We first treated these two cell lines with various concentrations of venetoclax (0–1,000 μM) in the presence/absence of ATO (3 µM). After incubation for 48 h, cell viability, apoptosis, and cell-cycle distribution were examined using the respective methods described in "Methods". As shown in Fig. 1a, b, the decrease in the level of cell viability was minimal to modest with the single-agent treatment of increasing concentrations of venetoclax in KG1 and KG1a cells. However, venetoclax substantially impaired the viability of these cells in a dose-dependent manner, when treated in combination with ATO (Fig. 1a, b).

We then measured the proportion of apoptotic cells using Annexin V and PI co-staining and flow cytometry analysis in these cells following treatment with increasing concentrations of venetoclax (0–1000 μM) in the presence/absence of ATO (3 µM). A consistent trend was noted, involving a significant rise in apoptosis rate with the combination of venetoclax and ATO in KG1 and KG1a cells; however, only a minimal to a modest degree of apoptosis was observed following venetoclax treatment alone (Fig. 1c, d). A noteworthy observation was that the cell death-enhancing effects of ATO were evident even with the lower doses of venetoclax (Fig. 1c, d). The combination effect generated by venetoclax and ATO on apoptosis was further examined by quantitative analysis of the dose–effect relationships based on the Chou–Talalay method [33]. As shown in Fig. 1e, f, there was a synergistic effect on apoptosis generated by the interaction between venetoclax and ATO; this was further indicated by CI values less than 1 in both KG1 and KG1a cells.

**Fig. 2:**
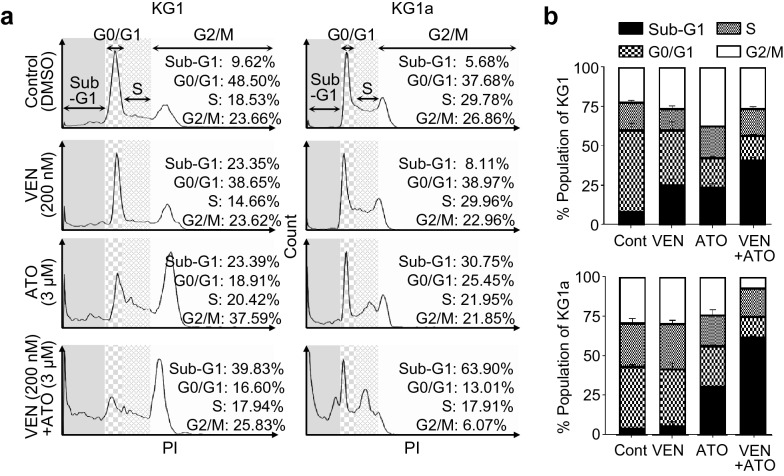
The combination of venetoclax and ATO disrupts the KG1 and KG1a cell cycles. **a**, **b**, Cell cycle analysis by flow cytometry after treatment with control (DMSO 0.1% v/v), venetoclax (200 nM), ATO (3 µM), or the combination of venetoclax (200 nM) and ATO (3 µM) for 48 h. Representative histograms of the cell cycles of the gated live KG1 and KG1a cells after treatment (**a**) and their summary data (**b**). Values were obtained from three independent experiments, and horizontal bars indicate mean ± s.d

Further analysis of cell cycle distribution revealed that the proportion of cells in the sub-G_1_ phase was significantly increased after the venetoclax and ATO combination treatment compared with either agent alone, indicating that the disrupted cell cycle caused by the combination treatment may be linked to the induction of apoptosis (Fig. 2a, b).

### The venetoclax and ATO combination preferentially induces apoptosis in primary CD34^+^ AML cells while sparing HSCs from healthy donors

**Fig. 3:**
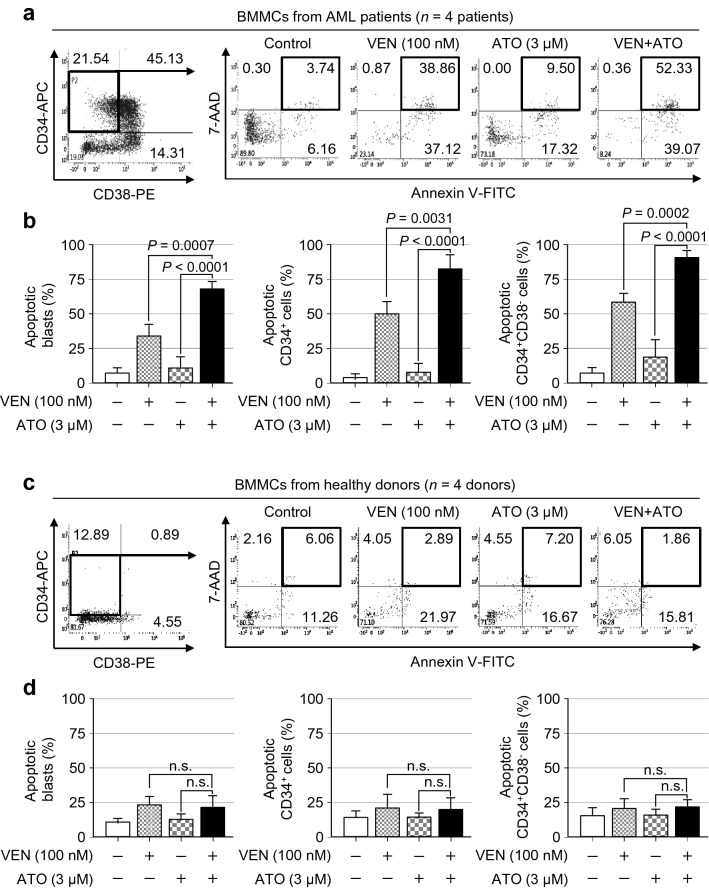
The venetoclax and ATO combination preferentially induces apoptosis of primary LSCs from AML patients while sparing healthy donor HSCs. **a**, **b**, Representative flow cytometric analysis (**a**) and summary data (**b**) of the percentage of Annexin V^+^7-AAD^+^ apoptotic cells after gating for CD34^+^CD38^−^ primary AML cells in the BMMCs of AML patients at diagnosis after treatment with venetoclax (100 nM), ATO (3 µM), or both in combination for 48 h in total mononuclear cells (**b**, far left), gated CD34^+^ cells (**b**, middle), or CD34^+^CD38^−^ cells (**b**, far right). Values were obtained from two independent experiments of *n* = 4 patients, and horizontal bars indicate mean ± s.d. *P values versus control treatment by two-tailed Mann–Whitney *U* test. n.s., not significant. **c**, **d**, Representative flow cytometric analysis (**c**) and summary data (**d**) of the percentage of Annexin V^+^7-AAD^+^ apoptotic cells after gating for CD34^+^CD38^−^ cells in the BMMCs of healthy donors following treatment with venetoclax (100 nM), ATO (3 µM), or both in combination for 48 h in total mononuclear cells (**d**, far left), gated CD34^+^ cells (**d**, middle), or CD34^+^CD38^−^ cells (**d**, far right). Values were obtained from two independent experiments of *n* = 4 healthy donors, and horizontal bars indicate mean ± s.d. *P values versus control treatment by two-tailed Mann–Whitney *U* test. n.s., not significant

To further examine whether ATO effectively promotes the venetoclax-induced apoptosis in primary AML LSC-like cells, we harvested diagnostic BMMCs or BMMCs at relapse from AML patients without any cytogenetic abnormalities. The clinical characteristics of the AML patients are summarised in Additional file 1: Table S1, S2. BMMCs from AML patients were treated with 100 nM of venetoclax in the presence/absence of 3 μM of ATO. After 48 h of incubation, the fraction of apoptotic cells was measured among the blast gate, CD34^+^ blasts, and CD34^+^CD38^−^ cells using Annexin V and 7-AAD staining and flow cytometry analysis. Representative flow cytometric plots of gated CD34^+^CD38^−^ primary AML cells after the single or combination treatment with venetoclax and ATO are shown in Fig. 3a and Additional file 2: Fig. S1.

With venetoclax treatment alone, the frequencies of Annexin V^+^7-AAD^+^ apoptotic cells in the mononuclear cell blast gate, CD34^+^ blasts, and CD34^+^CD38^−^ blast population were 33.93 ± 9.13%, 49.98 ± 9.31%, and 58.51 ± 6.42, respectively (Fig. 3b). After ATO treatment alone, the apoptotic cell fraction was 11.54 ± 7.51%, 7.91 ± 6.79%, and 19.1 ± 12.3% in the mononuclear cell blast gate, CD34^+^ blast, and CD34^+^CD38^−^ blast population, respectively (Fig. 3b). However, with the combination treatment of venetoclax and ATO, the apoptotic fraction was significantly increased to 68.00 ± 5.66% in the mononuclear cell blast gate (*P* = 0.0007 *vs* venetoclax alone; *P* < 0.0001 *vs* ATO alone) (Fig. 3b). The differences in the level of apoptosis remained significant when analyses were performed for gated CD34^+^ cells and CD34^+^CD38^−^ LSC-like cells (for CD34^+^ cells, *P* = 0.0031 *vs* venetoclax and *P* < 0.0001 *vs* ATO; for CD34^+^CD38^−^ blasts, *P* = 0.0002 *vs* venetoclax and *P* < 0.0001 *vs* ATO) (Fig. 3b). The augmented induction of apoptosis by the combination of venetoclax and ATO was observed not only in gated CD34^+^CD38^−^ blasts, but also in gated CD34^+^CD38^+^ or CD34^−^ cells of BMMCs at AML diagnosis (Fig. 3a, b and Additional file 3: Fig. S2), in addition to the BMMCs of relapsed AML patients (Additional file 4: Fig. S3).

Interestingly, as depicted in the representative flow cytometric plots of gated CD34^+^CD38^−^ cells after the single or combination treatment with venetoclax and ATO in the BMMCs of healthy donors (Fig. 3c and Additional file 2: Fig. S1), the combined effect of these agents on apoptosis was minimal in CD34^+^CD38^−^ cells as well as in the gated blasts, CD34^+^ cells of healthy BMMCs (Fig. 3d). These findings implied that the combination of venetoclax and ATO promotes induction of apoptosis preferentially in bulk AML cells and LSC-like cells while sparing HSCs.

### The venetoclax and ATO combination potentially activates the caspase-dependent mitochondrial apoptotic pathway

**Fig. 4:**
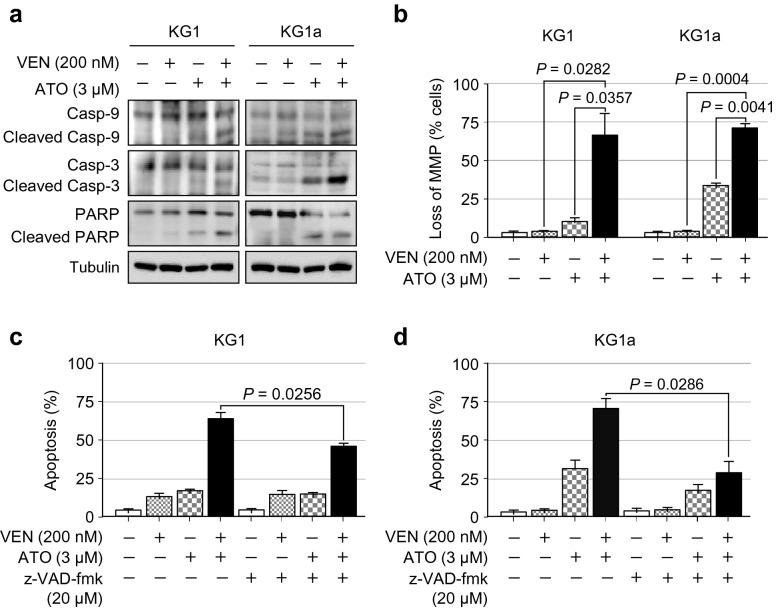
The combination of venetoclax and ATO promotes caspase-dependent apoptosis in KG1 and KG1a cells. **a**, Representative western blot analysis of the indicated proteins in KG1 and KG1a cells treated with venetoclax (200 nM), ATO (3 µM), or both in combination for 48 h. Similar results were obtained from three independent experiments. **b**, Summary data of MMP disruption, as assessed by flow cytometry, using the DePsipher Kit (Trevigen) in KG1 and KG1a cells treated with venetoclax (200 nM), ATO (3 µM), or both in combination for 48 h. Values were obtained from three independent experiments, and horizontal bars indicate mean ± s.d. *P values versus control treatment by two-tailed Mann–Whitney *U* test. n.s., not significant. **c**, **d**, Summary data of the percentage of apoptotic cells, as assessed by Annexin V/PI staining and flow cytometric analysis after treatment with or without the pan-caspase inhibitor z-VAD-fmk (20 µM) for 2 h prior to the addition of venetoclax (200 nM), ATO (3 µM), or both in combination for 48 h in KG1 (**c**) and KG1a (**d**) cells. Values were obtained from three independent experiments, and horizontal bars indicate mean ± s.d. *P values versus control treatment with or without z-VAD-fmk by two-tailed Mann–Whitney *U* test. n.s., not significant

We next evaluated the changes in caspase cleavage and MMP to unravel the mechanism of cell death involved in the combination of venetoclax and ATO in LSC-like cells. Compared with a single treatment, the combination of venetoclax and ATO resulted in an increase in the levels of cleaved caspase-9, cleaved caspase-3, and cleaved PARP (Fig. 4a).

To further investigate the cell death mechanism potentially triggered by the combination of venetoclax and ATO, MMP was analysed by flow cytometry using the DiOC_6_ probe. The venetoclax and ATO combination robustly induced a higher degree of MMP depolarisation compared with the extent of depolarisation induced by a single treatment with either agent in both KG1 and KG1a cells (Fig. 4b). Next, apoptotic status was analysed after the preincubation of these cells with pan-caspase inhibitor z-VAD-fmk (20 μM) for 2 h. As shown in Fig. 4c, d, z-VAD-fmk preincubation significantly alleviated apoptosis induction by combination treatment with venetoclax and ATO in both KG1 and KG1a cells. Collectively, these findings indicated that the synergistic increase in the rate of apoptosis by the venetoclax and ATO combination is, at least in part, attributable to the activation of the caspase-dependent mitochondrial apoptotic pathway.

### Mcl-1 protein is downregulated by adding ATO to venetoclax treatment in KG1 and KG1a cells

**Fig. 5:**
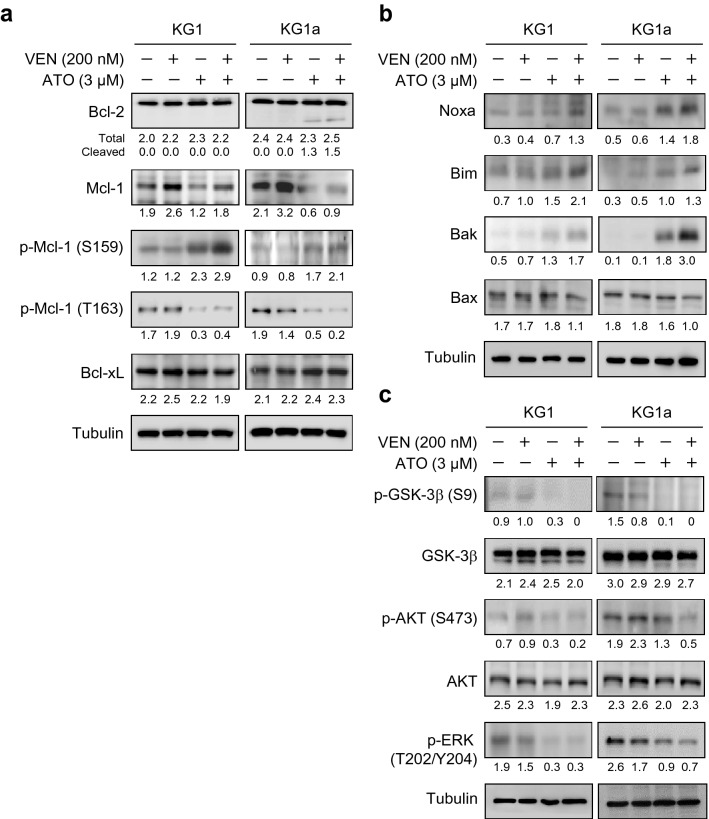
The venetoclax and ATO combination downregulates Mcl-1 through GSK-3β activation in KG1 and KG1a cells. **a–c**, Representative western blot analysis of the indicated proteins in KG1 and KG1a cells treated with venetoclax (200 nM), ATO (3 µM), or both in combination for 48 h. Similar results were obtained from three independent experiments

To elucidate the molecular mechanisms involved in the synergistic increase in apoptosis level with the combination of venetoclax and ATO, we next examined the effects of this combination on the Bcl-2 family members, which are critical regulators of apoptosis [9]. There were no apparent changes in the protein level of Bcl-2 following treatment with venetoclax in the presence/absence of ATO for 48 h in KG1 and KG1a cells (Fig. 5a). However, cleaved Bcl-2 was generated after treatment with ATO alone or in combination with venetoclax in KG1a cells, which was not observed in KG1 cells (Fig. 5a), suggesting the contextual differences between these cells with respect to the molecular alterations involving the cellular apoptosis machinery.

Next, we evaluated the changes in the Mcl-1 protein level. There was a noticeable elevation in the Mcl-1 protein level with venetoclax single treatment in both KG1 and KG1a cells (Fig. 5a). However, when these cells were treated with the combination of venetoclax and ATO, Mcl-1 protein levels were substantially downregulated in both these cell types (Fig. 5a). Remarkably, alleviation of the venetoclax-induced upregulation of Mcl-1 by ATO addition was conspicuous in KG1a cells (Fig. 5a).

To further elucidate the mechanism of Mcl-1 protein downregulation, we investigated the levels of Mcl-1 phosphorylation at Ser^159^ (p-Mcl-1 Ser^159^) as well as at Thr^163^ residue (p-Mcl-1 Thr^163^). In parallel with the downregulation of Mcl-1 protein, the level of p-Mcl-1 Ser^159^ was increased, whereas the level of p-Mcl-1 Thr^163^ was decreased following the combination treatment of venetoclax and ATO in both KG1 and KG1a cells (Fig. 5a). However, the change in the protein expression level of another anti-apoptotic member, Bcl-xL, was minimal after the combination treatment or either agent alone (Fig. 5a).

Notably, the protein level of Noxa, which is antagonised by Mcl-1 [35], was elevated after venetoclax and ATO combination treatment in both KG1 and KG1a cells (Fig. 5b). Likewise, the increase in the proapoptotic protein level of Bim, which forms a complex with Mcl-1 [9], was highest when these cells were treated with the combination of venetoclax and ATO (Fig. 5b). The protein level of Bak, which is a core regulator of mitochondrial outer membrane permeabilisation and apoptosis that is normally sequestered by Mcl-1 [9], was strongly induced upon combination treatment, especially in KG1a cells, while to a lesser extent with ATO single treatment (Fig. 5b). The change in the protein level of another proapoptotic effector Bax was minimal, implying that Bax may not be a major contributor to enhanced cell death with the venetoclax and ATO combination in these cells (Fig. 5b). Taken together, these findings indicated that Noxa and Bim may be untethered from Mcl-1-mediated binding through downregulation of Mcl-1, leading to the increased induction of apoptosis associated with Bak activation.

### The venetoclax and ATO combination activates GSK-3β in KG1 and KG1a cells

Since GSK-3β activation promotes p-Mcl-1 Ser^159^ leading to proteasomal degradation of Mcl-1 protein [9], we evaluated GSK-3β activity by measuring the level of GSK-3β phosphorylation at Ser^9^ (p-GSK-3β Ser^9^). Although the change in the total protein level of GSK-3β was minimal, the level of p-GSK-3β Ser^9^ was notably decreased after treatment with ATO. This was mediated by enhanced GSK-3β activity induced by adding ATO to venetoclax treatment in KG1 and KG1a cells (Fig. 5c). The phosphorylated form of AKT (Ser^473^) was diminished without significant changes in the level of total AKT by the combination treatment in both these cell types (Fig. 5c). The level of phosphorylated ERK (Thr^202^/Tyr^204^), which phosphorylates Mcl-1 at the Thr^163^ residue (p- Mcl-1 Thr^163^) and stabilises Mcl-1, was also reduced in KG1 and KG1a cells in response to treatment with the combination of venetoclax and ATO (Fig. 5c). These results signified that venetoclax-induced Mcl-1 upregulation is mitigated by ATO via GSK-3β activation-mediated increase in the level of p-Mcl-1 Ser^159^ and decrease in the level of p-Mcl-1 Thr^163^. This may subsequently lead to the proteasomal degradation and destabilsation of Mcl-1 protein.

### Ectopic Mcl-1 overexpression alleviates the venetoclax and ATO combination-induced apoptosis

**Fig. 6:**
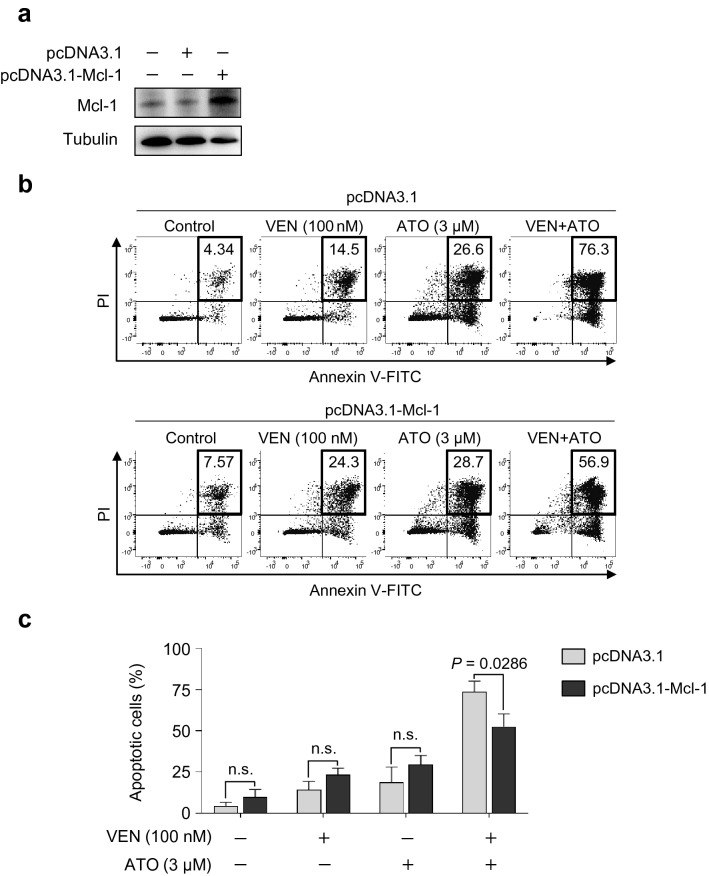
Mcl-1 overexpression alleviates apoptosis induced by the combination of venetoclax and ATO in KG1a cells. **a**, Western blot analysis of indicated proteins in KG1a cells treated with venetoclax (200 nM), ATO (3 µM), or both in combination for 48 h after transfection with pcDNA3.1 control or pcDNA3.1-Mcl-1 vector. **b**, **c**, Representative flow cytometric analysis (**b**) and summary data (**c**) of the percentage of Annexin V^+^PI^+^ apoptotic cells in KG1a cells transfected with pcDNA3.1 control or pcDNA3.1-Mcl-1 vector after treatment with venetoclax (100 nM), ATO (3 µM), or both in combination for 48 h. Values were obtained from two independent experiments, and horizontal bars indicate mean ± s.d. *P values versus control treatment by two-tailed Mann–Whitney *U* test. n.s., not significant

To further elucidate the role of Mcl-1 downregulation in the venetoclax and ATO combination-induced synergistic cell death, Mcl-1 overexpression was ectopically induced in KG1a cells (Fig. 6a), as described in "Methods". Briefly, KG1a cells were transfected with pcDNA3.1 control or pcDNA3.1-Mcl-1 vector, and the level of apoptosis was examined with the Annexin V and PI staining after the single or combined treatment with venetoclax and ATO (Fig. 6b). The proportion of apoptotic cells was significantly diminished after the combination treatment in KG1a cells upon Mcl-1 overexpression (52.80 ± 6.59%), compared with the combination treatment in control KG1a cells without Mcl-1 overexpression (72.50 ± 5.47%, *P* = 0.0286) (Fig. 6b,c). These findings indicate that downregulation of Mcl-1 is critically involved in the synergistic increase in the level of apoptosis in AML LSC-like cells treated with a combination of venetoclax and ATO.

### Venetoclax and ATO combination triggers robust DNA damage response

**Fig. 7:**
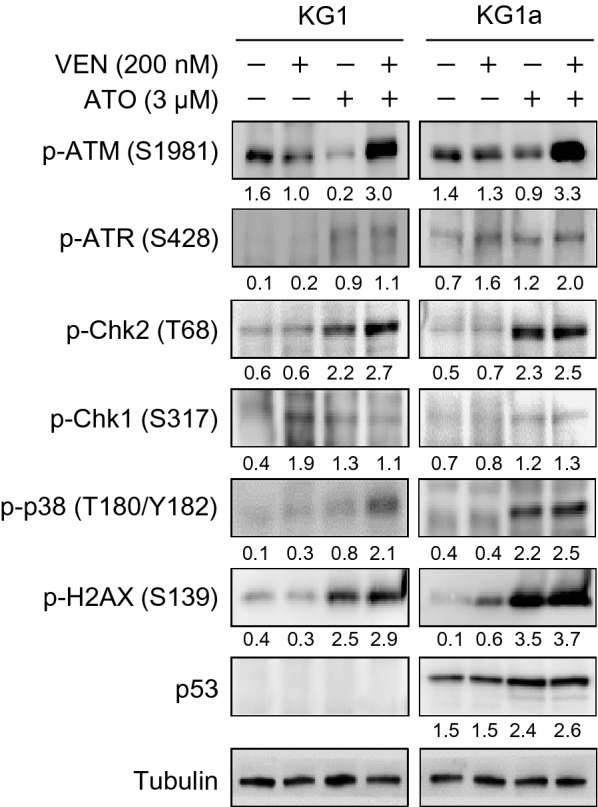
The venetoclax and ATO combination triggers robust DNA damage response. Representative western blot analysis of the indicated proteins in KG1 and KG1a cells treated with venetoclax (200 nM), ATO (3 µM), or both in combination for 48 h. Similar results were obtained from three independent experiments

The findings above led us to explore the changes in the level of proteins involved in the DNA damage response after venetoclax and ATO treatment alone or in combination in KG1 and KG1a cells. As shown in Fig. 7, the combination of venetoclax and ATO robustly increased the levels of p-ATM (Ser^1981^) and p-H2AX (Ser^139^) compared with the single treatment of either agent alone. In parallel, Chk2 phosphorylation at Thr^68^ residue was markedly augmented with the combination treatment (Fig. 7). Phosphorylation of p38 mitogen-activated protein kinase (MAPK) was also augmented with the combined treatment of venetoclax and ATO compared with the treatment of either agent alone (Fig. 7), signifying the robust induction of apoptosis by the combination treatment in these cells. However, compared with the phosphorylation of ATM and Chk2, the phosphorylation of ATR (Ser^428^) and Chk1(Ser^317^) were modest following the combination treatment (Fig. 7). Changes in p53 protein expression levels were not apparent in both KG1 and KG1a cells (Fig. 7). Taken together, these findings implied that p53-independent DNA damage response, especially the p-ATM (Ser^1981^) and p-Chk2 (Thr^68^) pathway, is associated with enhanced induction of apoptosis by the combination of venetoclax and ATO.

## Discussion

**Fig. 8:**
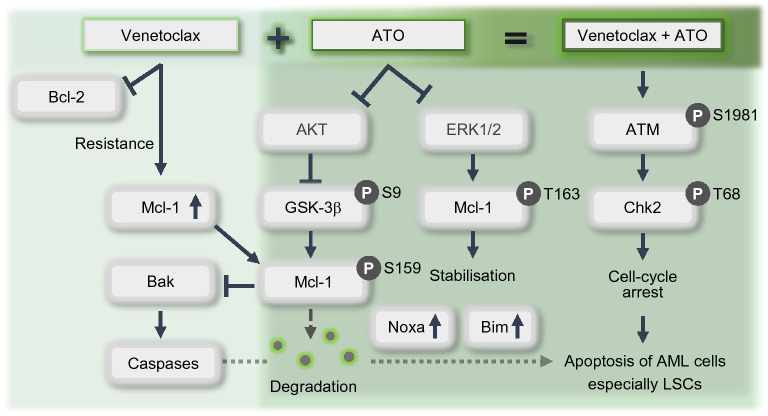
Proposed mechanism of synergistic action between venetoclax and ATO. Schematic diagram depicting the synergism between venetoclax and ATO in LSC-like leukaemia cells and primary LSCs. Bcl-2 inhibition by venetoclax in LSC-like leukaemia cells leads to the upregulation of Mcl-1, which confers resistance to venetoclax. However, upon combination treatment with venetoclax and ATO, AKT is attenuated and subsequently impairs GSK-3β phosphorylation at Ser9 and activates GSK-3β. This leads to the phosphorylation of Mcl-1 at Ser159, triggering Mcl-1 degradation. The levels of Noxa and Bim, which are normally sequestered by Mcl-1, are elevated upon Mcl-1 degradation and promote caspase-dependent apoptosis associated with Bak activation. In parallel, ERK downregulation attenuates Mcl-1 phosphorylation at Thr163, leading to Mcl-1 destabilisation. The venetoclax and ATO combination also induces the phosphorylation of ATM at Ser1981 and Chk2 at Thr68, promoting cell cycle arrest associated with a robust DNA damage response and apoptosis

In the present study, we demonstrated that a combination of venetoclax and ATO synergistically promotes apoptosis in LSC-like leukaemia cells and induces apoptosis preferentially in primary LSCs from AML patients, concomitantly sparing HSCs from healthy donors. We further revealed that ATO mitigates venetoclax-induced upregulation of Mcl-1. The synergistic effects of this venetoclax and ATO combination are mediated by attenuated AKT and ERK with GSK-3β activation, consequently triggering Mcl-1 destabilisation and degradation, via the activation of caspase-dependent apoptotic cell death and untethering of Noxa and Bim from Mcl-1, and Bak activation, associated with a strong DNA damage response (Fig. 8).

Better understanding of the mechanisms that mediate synergism between venetoclax and ATO in AML cells other than Mcl-1 is still needed. Although other mechanisms, such as the alterations in the fatty acid metabolism [36], mitochondrial oxidative phosphorylation [37], and electron transport chain complexes [38], may be involved in the synergistic interaction, Mcl-1 inhibition was evident to be importantly associated with the synergistic cell death in AML cells induced with venetoclax and ATO combination in this study. Therefore, our data reinforce the prevailing concept of Mcl-1 as an important resistance factor to venetoclax and confirm prior studies delineating Mcl-1 as a mediator of the differential responsiveness to ATO between APL and non-APL cells [31]. It was proposed that ATO distinctly downregulates Mcl-1 via GSK-3β activation in APL cells; however, this was not the case in non-APL cells, where the additional modulation of the Mcl-1 level was necessary to sensitise non-APL cells to ATO-induced apoptosis [31]. Supporting this notion, we revealed that ATO activates GSK-3β in KG1 and KG1a cells when combined with venetoclax, which was associated with attenuated AKT and enhanced phosphorylation of Mcl-1 at Ser^159^, subsequently leading to Mcl-1 degradation. We also showed that the combination reduces p-ERK levels and diminishes Mcl-1 phosphorylation at Thr^163^, which is required for Mcl-1 stabilisation [39]. These results jointly indicated that ATO-induced downregulation of the increased Mcl-1 level in venetoclax-treated KG1 and KG1a cells could occur via similar mechanisms to those employed in ATO-treated APL cells.

Venetoclax treatment combined with the direct inhibition of Mcl-1 by a lentiviral BH3-expressing vector is efficacious in overcoming venetoclax resistance in xenograft models of AML [40]. Moreover, the addition of the selective Mcl-1 inhibitor S63845 [41, 42], AMG 176, or AM-8621 [43] enhances the sensitivity to venetoclax in AML cell lines as well as in primary AML cells. Indeed, these synergistic effects are also observed in AML progenitor cells [33]. However, similar to other anti-apoptotic proteins of the Bcl-2 family, Mcl-1 plays a vital role in the survival of various cells of normal tissues, including cardiac and hepatic cells [44, 45], pluripotent stem cells [46], and neurons [47]. The development of selective Mcl-1 inhibitors is seriously hampered by safety concerns and technical drawbacks in targeting Mcl-1 [25]. Therefore, these challenges require extensive validation of observed results before direct Mcl-1 inhibitors become easily accessible and applicable in routine clinical practice [22].

A range of compounds indirectly causes a decline in the cellular level of Mcl-1 and its key signalling molecules. XPO1-selective inhibitor selinexor [48, 49], autophagy inhibitor spautin-1 [50], tyrosine kinase inhibitor sorafenib [51, 52], survivin inhibitor YM155 [53], and CDK9 inhibitors [54, 55] are the representative compounds. Furthermore, MAPK signalling regulation by p53 activation [56], inhibitors of Nedd8-activating enzyme [57], as well as MEK [58], MDM2 [59], glutaminase [60], and pan-RAF [61] inhibitors have also been shown to reduce the level of Mcl-1. Although preclinical studies have demonstrated these agents’ efficacy, only a few of them are being investigated in clinical trials. It is anticipated that there would be a prolonged period preceding the application of safe and tolerable modulators of Mcl-1. Hence, it is extremely crucial to identify readily available and clinically applicable agents that inhibit Mcl-1 to improve the therapeutic efficacy of venetoclax. In this regard, the venetoclax and ATO combination may serve as a feasible and safe option for treating AML patients, as they possess a relatively optimal tolerability profile and non-overlapping harmful effects [62].

It has been recently recognised that the combination of venetoclax and ATO induces apoptosis in *NPM1*-mutated OCI-AML3 cells, which constitute the cell line inherently resistant to venetoclax [63]. OCI-AML3 cells express high levels of Mcl-1, which has been shown to be further upregulated upon treatment with venetoclax [56]. However, the *NPM1* mutation in OCI-AML3 cells confers sensitivity to ATO even in the absence of venetoclax [64], and the changes in the level of Mcl-1 underlying the proposed synergism have not yet been demonstrated. Our data support the synergistic antileukaemic effects of venetoclax and ATO and provide mechanistic insights that the synergism between venetoclax and ATO is mediated via ATO-mediated Mcl-1 downregulation.

We demonstrated that the combination of venetoclax and ATO spares HSCs from healthy donors while concomitantly promoting apoptosis in LSC-like cells. Homeostasis of healthy HSCs is regulated by oxidative phosphorylation (OXPHOS)-driven regeneration and glycolysis-mediated quiescence [65]. This balance is dysregulated in AML LSCs, as LSCs preferentially depend on mitochondrial OXPHOS to a greater degree than on glycolysis [7]. Considering that ATO activates quiescent HSCs and LSCs [66], the simultaneous inhibition of increased mitochondrial OXPHOS by venetoclax and unleashing LSC quiescence by ATO could have cooperatively sensitised LSCs to the combination treatment while sparing healthy HSCs. Indeed, no appreciable therapeutic effects of ATO as a single-agent in non-APL AML cells have been demonstrated to date [40, 67, 68], where we also observed the modest induction of apoptosis by ATO treatment alone. Given that ATO is relatively safe and tolerable in the current clinical settings [69], our data may provide the rationale for future preclinical and clinical trials for prolonged infusion of ATO in combination with venetoclax, which may generate an opportunity to capture a larger proportion of leukaemic cells, including LSCs, which asynchronously enter the S phase [66].

We highlighted the synergism between venetoclax and ATO in mutant p53-expressing KG1 and KG1a cell lines. There were minimal changes in p53 protein expression when these cells were treated with either venetoclax and ATO alone or in combination. Although p53 activation and Bcl-2 inhibition have been shown to reciprocally overcome resistance to each other in primary AML cells, at least in part, through the negative regulation of Mcl-1 by p53 [56], our results showed that the benefits of the venetoclax and ATO combination treatment in promoting apoptosis are efficacious irrespective of the p53 status. Therefore, our study may provide an opportunity for improving outcomes in patients harbouring p53-mutated AML, which is marked by an extremely poor prognosis [2, 70]. Moreover, our proposed concept may not be limited to AML. Other haematological malignancies expressing high levels of both Bcl-2 and Mcl-1, such as multiple myeloma [71], may also benefit from the venetoclax and ATO combination, which needs to be validated in future research.

## Conclusions

In summary, our study elucidates the synergism between venetoclax and ATO in promoting LSC apoptosis by inhibiting AKT and ERK, leading to GSK-3β activation and Mcl-1 destabilisation, which will support future preclinical and clinical trials evaluating the suitability of this combination for treating AML. This finding may lead to exciting prospects for a chemotherapy-free approach, especially for AML patients who are vulnerable and not strong enough to undergo high-intensity chemotherapy.

## Supplementary Information

**Additional file 1: Table S1**. Baseline patient characteristics of primary AML samples at diagnosis.** Table S2.** Patient characteristics of primary AML samples at relapse.

**Additional file 2: Fig. S1.** The venetoclax and ATO combination preferentially induces apoptosis of primary LSCs from AML patients while sparing healthy donor HSCs. Representative flow cytometric analysis of the unstained control for Fig. 3 in the BMMCs of AML patients at diagnosis (left panel) or healthy donors (right panel).

**Additional file 3: Fig. S2.** The venetoclax and ATO combination promotes apoptosis of gated CD34^+^CD38^+^ or CD34^−^ primary LSCs from AML patients. Representative flow cytometric analysis of the percentage of Annexin V^+^7-AAD^+^ apoptotic cells after gating for CD34^+^CD38^+^ (above panels) or CD34^−^ (below panel) primary AML cells in the BMMCs of AML patients at diagnosis after treatment with venetoclax (100 nM), ATO (3 μM), or both in combination for 48 h.

**Additional file 4: Fig. S3.** The venetoclax and ATO combination promotes apoptosis of primary LSCs from relapsed AML patients. a, b, Representative flow cytometric analysis (a-c) and summary data (d) of the percentage of Annexin V^+^7-AAD^+^ apoptotic cells after gating for CD34^+^CD38^−^ (a), CD34^+^CD38^+^ (b), or CD34^−^ (c) primary AML cells in the BMMCs of relapsed AML patients after treatment with venetoclax (100 nM), ATO (3 μM), or both in combination for 48 h in total mononuclear cells (d, far left), gated CD34^+^ cells (d, middle), or CD34^+^CD38^−^ cells (d, far right). Values were obtained from two independent experiments of *n *= 4 patients, and horizontal bars indicate mean ± s.d. *P values versus control treatment by two-tailed Mann–Whitney *U *test.

## Data Availability

Source data and materials in this study are available from the corresponding author upon reasonable request.

## Abbreviations

AML: Acute myeloid leukaemia
APL: Acute promyelocytic leukaemia
ATRA: All-trans retinoic acid
BM: Bone marrow
BMMC: BM mononuclear cell
DMSO: Dimethyl sulfoxide
ATO: Arsenic trioxide
HSC: Haematopoietic stem cell
HSCT: Haematopoietic stem cell transplantation
LSC: Leukaemic stem cell
MAPK: Mitogen-activated protein kinase
MMP: Mitochondrial membrane potential
OXPHOS: Oxidative phosphorylation
PARP: Poly(ADP-ribose) polymerase
PI: Propidium iodide
RIPA: Radioimmunoprecipitation
ROS: Reactive oxygen species
