# Supplementary material for: Arsenic trioxide synergistically promotes the antileukaemic activity of venetoclax by downregulating Mcl-1 in acute myeloid leukaemia cells
Source: Exp Hematol Oncol. 2021 Apr 15;10:28. doi: 10.1186/s40164-021-00221-6 (PMC8051086; doi:10.1186/s40164-021-00221-6)
Supplement: Supplementary file 1 — Additional file 1: Table S1. Baseline patient characteristics of primary AML samples at diagnosis. Table S2. Patient characteristics of primary AML samples at relapse. [file 40164_2021_221_MOESM1_ESM.docx]

**Table S1. Baseline patient characteristics of primary AML samples at diagnosis**

| Parameters | Median (range) |
| --- | --- |
| Age (years) | 52.5 (44­–65) |
| WBC, ×10^9^/L | 20.6 (2.4­–69.5) |
| LDH, IU/L | 463 (310­–749) |
| Bone marrow blasts, % | 70.7 (52.0­–86.3) |
| Peripheral blood blasts, % | 56.5 (31­–67) |

Abbreviations: AML, acute myeloid leukemia; WBC, white blood cells; LDH, lactate dehydrogenase.

**Table S2. Patient characteristics of primary AML samples at relapse**

| Parameters | Median (range) or  number of patients |
| --- | --- |
| Age (years) | 67.0 (35­–76) |
| Remission status prior to relapse |  |
| 1^st^ CR | 3 |
| 2^nd^ CR | 1 |
| Bone marrow blast, % | 82.3 (23.5­–93.4) |

Abbreviations: AML, acute myeloid leukemia; CR, complete remission.
