# Supplementary material for: Arsenic trioxide synergistically promotes the antileukaemic activity of venetoclax by downregulating Mcl-1 in acute myeloid leukaemia cells
Source: Exp Hematol Oncol. 2021 Apr 15;10:28. doi: 10.1186/s40164-021-00221-6 (PMC8051086; doi:10.1186/s40164-021-00221-6)
Supplement: Supplementary file 2 — Additional file 2: Fig. S1. The venetoclax and ATO combination preferentially induces apoptosis of primary LSCs from AML patients while sparing healthy donor HSCs. Representative flow cytometric analysis of the unstained control for Fig. 3 in the BMMCs of AML patients at diagnosis (left panel) or healthy donors (right panel). [file 40164_2021_221_MOESM2_ESM.pdf]

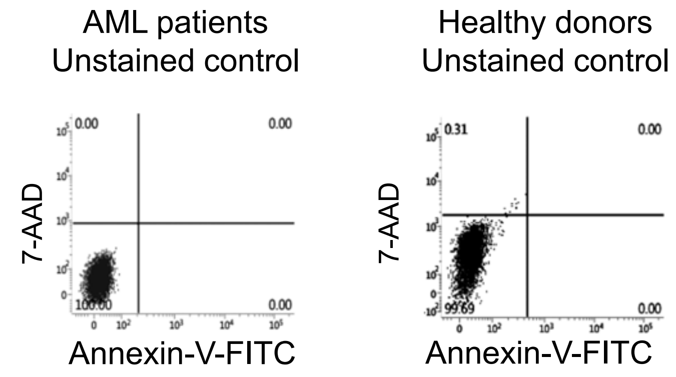

Additional file 2: Fig. S1. The venetoclax and ATO combination preferentially induces apoptosis of primary LSCs from AML patients while sparing healthy donor HSCs.

Representative flow cytometric analysis of the unstained control for Fig. 3 in the BMMCs of AML patients at diagnosis (left panel) or healthy donors (right panel).
