# Supplementary material for: Arsenic trioxide synergistically promotes the antileukaemic activity of venetoclax by downregulating Mcl-1 in acute myeloid leukaemia cells
Source: Exp Hematol Oncol. 2021 Apr 15;10:28. doi: 10.1186/s40164-021-00221-6 (PMC8051086; doi:10.1186/s40164-021-00221-6)
Supplement: Supplementary file 3 — Additional file 3: Fig. S2. The venetoclax and ATO combination promotes apoptosis of gated CD34+CD38+ or CD34− primary LSCs from AML patients. Representative flow cytometric analysis of the percentage of Annexin V+7-AAD+ apoptotic cells after gating for CD34+CD38+ (above panels) or CD34− (below panel) primary AML cells in the BMMCs of AML patients at diagnosis after treatment with venetoclax (100 nM), ATO (3 μM), or both in combination for 48 h. [file 40164_2021_221_MOESM3_ESM.pdf]

Figure S2

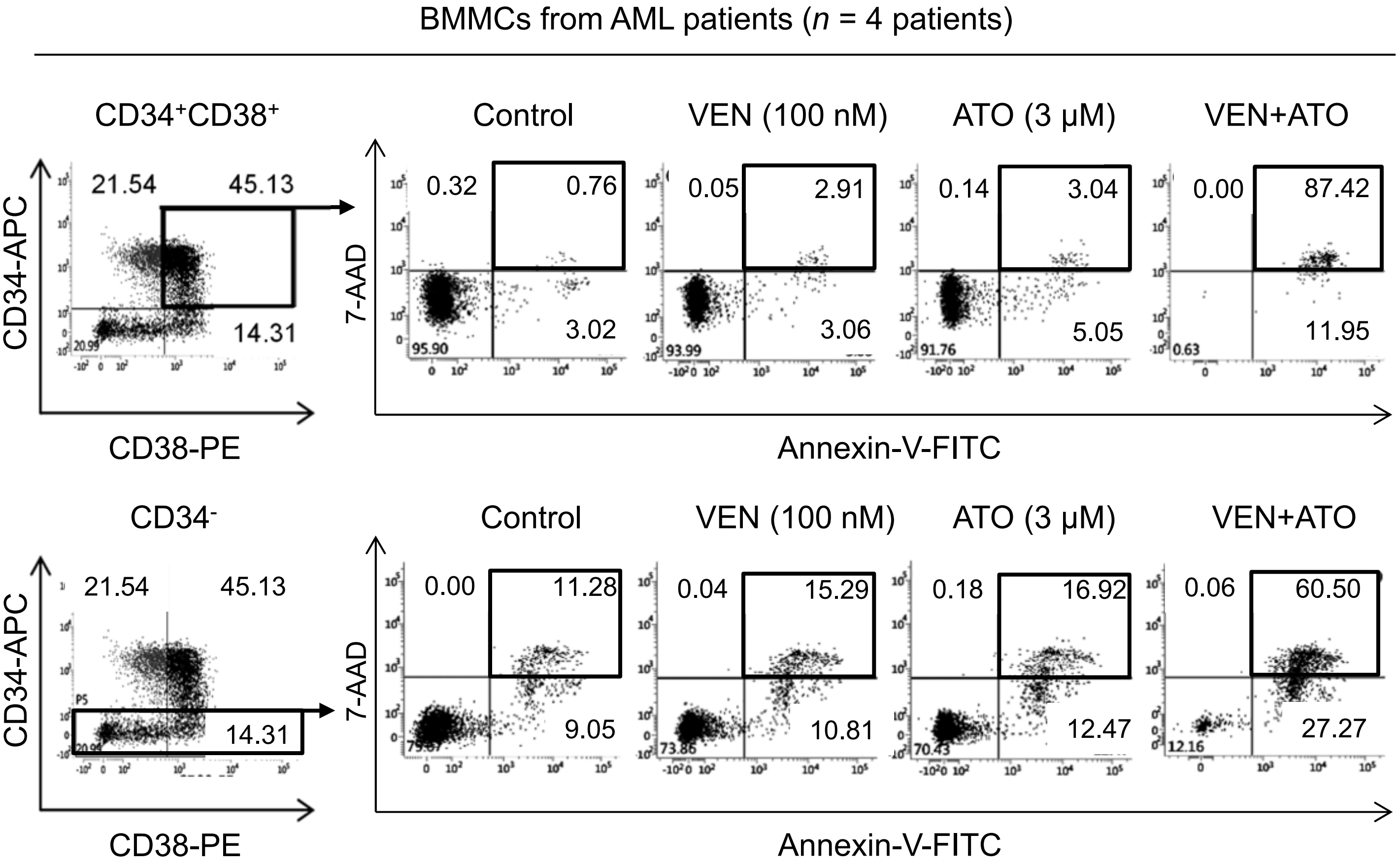

Additional file 3: Fig. S2. The venetoclax and ATO combination promotes apoptosis of gated CD34$^+$CD38$^+$ or CD34$^-$ primary LSCs from AML patients.

Representative flow cytometric analysis of the percentage of Annexin V$^+$7-AAD$^+$ apoptotic cells after gating for CD34$^+$CD38$^+$ (above panels) or CD34$^-$ (below panel) primary AML cells in the BMMCs of AML patients at diagnosis after treatment with venetoclax (100 nM), ATO (3 μM), or both in combination for 48 h.
