# Supplementary material for: Arsenic trioxide synergistically promotes the antileukaemic activity of venetoclax by downregulating Mcl-1 in acute myeloid leukaemia cells
Source: Exp Hematol Oncol. 2021 Apr 15;10:28. doi: 10.1186/s40164-021-00221-6 (PMC8051086; doi:10.1186/s40164-021-00221-6)
Supplement: Supplementary file 4 — Additional file 4: Fig. S3. The venetoclax and ATO combination promotes apoptosis of primary LSCs from relapsed AML patients. a, b, Representative flow cytometric analysis (a-c) and summary data (d) of the percentage of Annexin V+7-AAD+ apoptotic cells after gating for CD34+CD38− (a), CD34+CD38+ (b), or CD34− (c) primary AML cells in the BMMCs of relapsed AML patients after treatment with venetoclax (100 nM), ATO (3 μM), or both in combination for 48 h in total mononuclear cells (d, far left), gated CD34+ cells (d, middle), or CD34+CD38− cells (d, far right). Values were obtained from two independent experiments of n = 4 patients, and horizontal bars indicate mean ± s.d. *P values versus control treatment by two-tailed Mann–Whitney U test. [file 40164_2021_221_MOESM4_ESM.pdf]

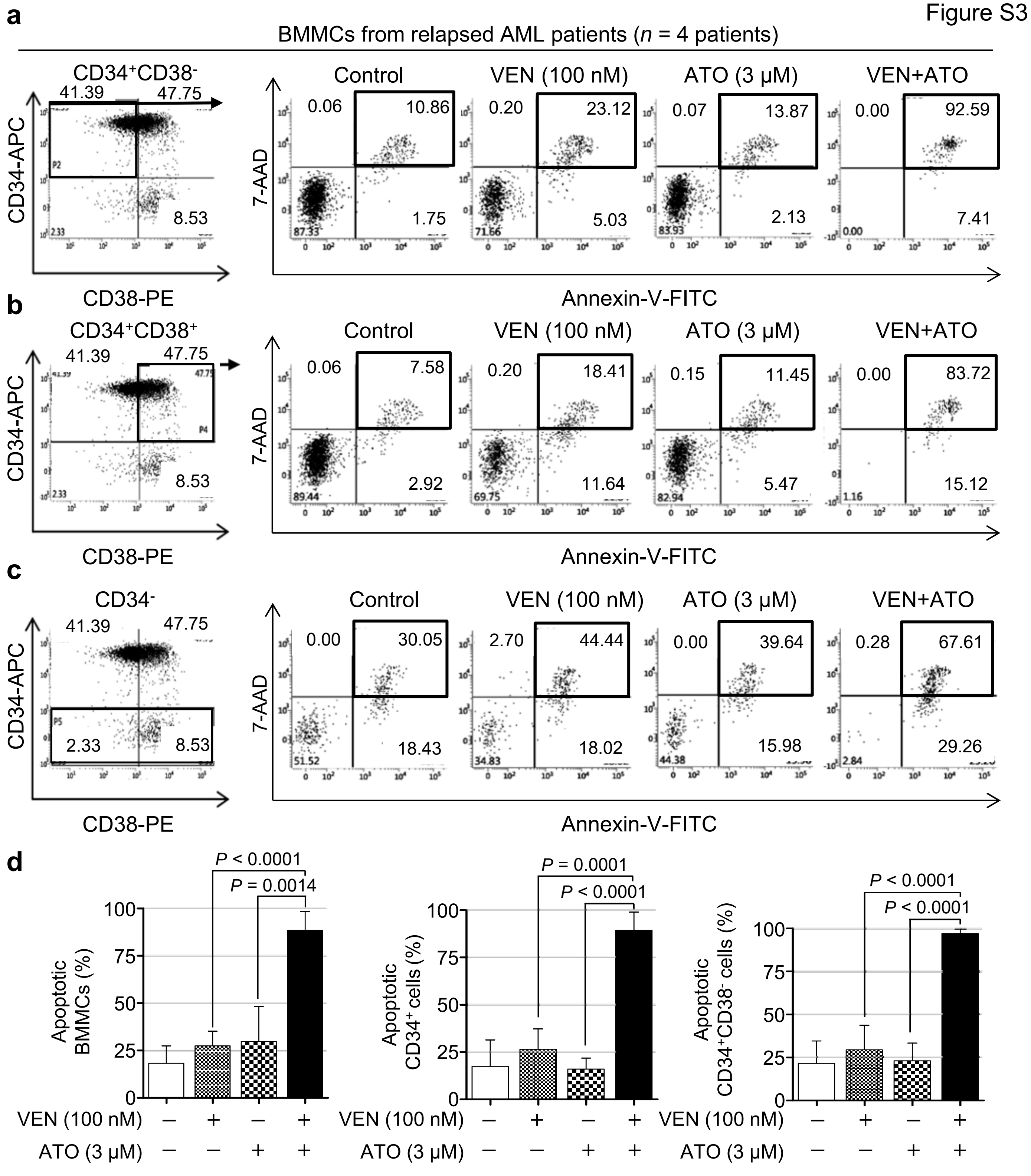

Additional file 4: Fig. S3. The venetoclax and ATO combination promotes apoptosis of primary LSCs from relapsed AML patients.

a, **b**, Representative flow cytometric analysis (**a-c**) and summary data (**d**) of the percentage of Annexin V$^+$7-AAD$^+$ apoptotic cells after gating for CD34$^+$CD38$^-$ (**a**), CD34$^+$CD38$^+$ (**b**), or CD34$^-$ (**c**) primary AML cells in the BMMCs of relapsed AML patients after treatment with venetoclax (100 nM), ATO (3 μM), or both in combination for 48 h in total mononuclear cells (**d**, far left), gated CD34$^+$ cells (**d**, middle), or CD34$^+$CD38$^-$ cells (**d**, far right). Values were obtained from two independent experiments of n = 4 patients, and horizontal bars indicate mean ± s.d. *P values versus control treatment by two-tailed Mann–Whitney U test.
